# A deep tabular data learning model predicting cisplatin sensitivity identifies BCL2L1 dependency in cancer

**DOI:** 10.1016/j.csbj.2023.01.020

**Published:** 2023-01-16

**Authors:** Ahmad Nasimian, Mehreen Ahmed, Ingrid Hedenfalk, Julhash U. Kazi

**Affiliations:** a Division of Translational Cancer Research, Department of Laboratory Medicine, Lund University, Lund, Sweden; b Lund Stem Cell Center, Department of Laboratory Medicine, Lund University, Lund, Sweden; c Division of Oncology, Department of Clinical Sciences Lund, Lund University and Skåne University Hospital, 223 81 Lund, Sweden

**Keywords:** WNT/β-catenin, XGBoost, Random Forest, Elastic net, Ovarian cancer, BCL-XL

## Abstract

Cisplatin, a platinum-based chemotherapeutic agent, is widely used as a front-line treatment for several malignancies. However, treatment outcomes vary widely due to intrinsic and acquired resistance. In this study, cisplatin-perturbed gene expression and pathway enrichment were used to define a gene signature, which was further utilized to develop a cisplatin sensitivity prediction model using the TabNet algorithm. The TabNet model performed better (>80 % accuracy) than all other machine learning models when compared to a wide range of machine learning algorithms. Moreover, by using feature importance and comparing predicted ovarian cancer patient samples, BCL2L1 was identified as an important gene contributing to cisplatin resistance. Furthermore, the pharmacological inhibition of BCL2L1 was found to synergistically increase cisplatin efficacy. Collectively, this study developed a tool to predict cisplatin sensitivity using cisplatin-perturbed gene expression and pathway enrichment knowledge and identified BCL2L1 as an important gene in this setting.

## Graphical Abstract

**Figure:**
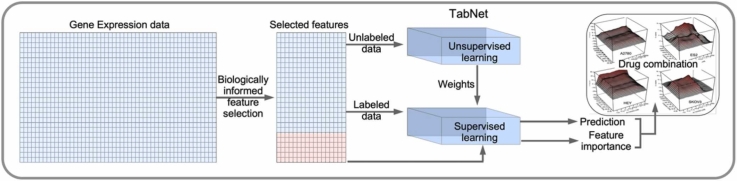

## 1 Introduction

Cisplatin and its analog platinum chemotherapeutic agents are nonspecific antitumor drugs used to treat a wide variety of cancers in clinical practice. Although all platinum-centered analogs function by binding DNA, the effectiveness of individual analogs is cancer subtype-specific. Cisplatin, which is the base drug and widely used in the treatment of several cancers, displays differential responses due to intrinsic and acquired resistance.

Intracellular cisplatin accumulation correlates with acquired resistance, which is dependent on the influx and efflux of the drug [1]. Cisplatin enters cells through passive diffusion and transporter proteins. The well-studied cisplatin transporter SLC31A1 (also known as CTR1) is a high-affinity copper influx transporter [2]. SLC31A2 (CTR2), another copper transporter, shares considerable structural similarity to SLC31A1 but is not involved in platinum uptake. In contrast, expression of SLC31A2 reduced cellular platinum accumulation, suggesting a role in platinum efflux [3]. Two copper-transporting P-type ATPases, ATP7A and ATP7B, also act as platinum efflux transporters [4], [5]. Thus, the loss of SLC31A1 or gain of SLC31A2, ATP7A, and ATP7B expression impairs cellular cisplatin accumulation and contributes to cisplatin resistance [6]. Other cisplatin transporters that play tissue-specific roles include SLC22A1 (OCT1), SLC22A2 (OCT2), and SLC47A1 (MATE1) [7], [8], [9]. While SLC22A1 and SLC22A2 increase cisplatin uptake, SLC47A1 serves as an efflux transporter. Two other efflux transporters are adenosine triphosphate (ATP)-binding cassette (ABC) multidrug transporters ABCC1 (MRP1) and ABCC2 (MRP2) [10], [11], [12].

In an aqueous environment, chloride ions (Cl^-^) in cisplatin are replaced by water molecules. The resulting electrophilic molecule is considered active cisplatin and can react with nucleophilic groups on DNA and sulfhydryl groups on proteins [13]. Such type of cross-linking causes DNA damage, cell cycle arrest, and apoptosis induction. The tripeptide thiol glutathione (GSH) is a potent nucleophile that can bind to platinum; the complex is then pumped out by ABCC2 [14], [15]. Therefore, cells with higher GSH levels can exhibit cisplatin resistance. Cysteine-rich protein metallothionein (MT) and glutathione transferase P1 (GSTP1) bind and inactivate cisplatin, thereby also resulting in cisplatin resistance [16], [17].

In response to cisplatin-mediated DNA crosslinking, cells activate multiple DNA repair pathways. If the damage is repairable, cells undergo cell cycle arrest in the S and G2 phases to allow the repair system to restore DNA integrity and prevent abnormal mitoses [18]. However, if the damage is beyond repair, cells activate the apoptosis pathway. It has been shown that the strong activity of the nucleotide excision repair system, which is composed of around 20 proteins, including ERCC1, mediates cisplatin resistance. The expression of ERCC1 has been reported to be negatively correlated with cisplatin response [19]. Furthermore, loss-of-function TP53 mutations can significantly lower cisplatin treatment efficacy [20].

Expression levels of antiapoptotic BCL2-family proteins such as BCL2, BCL-XL, and MCL1, as well as reactivation of the PI3K/AKT and MAP-kinase pathways, negatively correlate with cisplatin response [18]. Hence, as discussed above, cells can utilize a variety of mechanisms to defend against cisplatin effects. However, there may be additional factors contributing to cisplatin resistance that have yet to be identified.

Considering the complexity of cisplatin’s interactions with the cellular environment and its effectiveness, several studies have recently attempted to build cisplatin response prediction models based on specific gene signatures. Using a 15-gene signature (BARD1, BCL2, BCL2L1, CDKN2C, FAAP24, FEN1, MAP3K1, MAPK13, MAPK3, NFKB1, NFKB2, SLC22A5, SLC31A2, TLR4, and TWIST1), a support vector machine (SVM), and data from The Cancer Genome Atlas (TCGA) patients with bladder, ovarian and colorectal cancer, Mucaki *et al.* achieved 55–71 % accuracy in predicting cisplatin response [21]. Moreover, using lung cancer cell line data and SVM, Gao *et al.* identified a nine-gene signature (PLXNC1, KIAA0649, SPTBN4, SLC14A2, F13A1, COL5A1, SCN2A, PLEC, and ALMS1) that can predict cisplatin sensitivity [22]. Furthermore, while Shannon *et al.* identified four genes (CYTH3, GALNT3, S100A14, and ERI1) [23], Sui *et al.*, applying a regularized logistic regression model to multi-omics data, identified six genes (FOXA2, BATF3, SIX1, HOXA1, IRF5, and ZBTB38) associated with *in vitro* cisplatin sensitivity [24]. Notably, none of the signature genes from these four studies overlap, indicating that cisplatin sensitivity can be predicted using different gene signatures. Nevertheless, these works provide useful insights into the prediction of cisplatin response for future research. In this study, a drug-perturbed gene signature and a recently developed tabular deep learning framework, TabNet, were employed to predict cisplatin sensitivity. Deregulated pathway information was incorporated to identify the gene signature, and then identify key proteins that could be considered for combinatorial therapy to overcome resistance.

## 2 Results

### 2.1 Cisplatin treatment induces WNT/β-catenin signaling

**Fig. 1:**
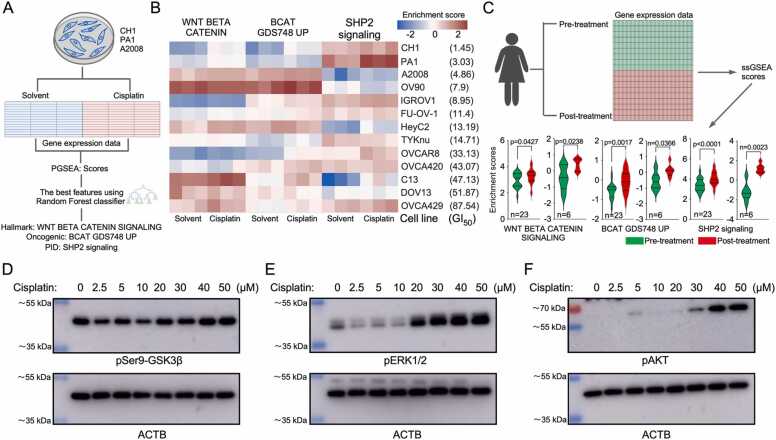
Enrichment of WNT/β-catenin pathway. (A) Gene expression data from three cisplatin-sensitive cell lines were treated with solvent or cisplatin before collecting mRNA for microarray analysis. PGSEA scores were calculated for all replicates of the three cell lines (nine in total). Using a random forest classifier, the most relevant pathways that explain cisplatin response were determined. (B) All cell lines were analyzed using PGSEA, and the scores were analyzed by hierarchically‐clustered heatmap (seaborn.clustermap). GI_50_ values were adopted from Miow *et al*. [25]. (C) Data from two ovarian cancer patient cohorts were analyzed via ssGSEA. Scores for specific pathways were compared. (D‐F) Cells were treated with different concentrations of cisplatin for 24 h before lysis. Lysates were used for SDS-PAGE separation following western blotting using respective antibodies.

Cisplatin is known to activate several signaling pathways regulating various cellular events [18]. To understand how cisplatin regulates cell signaling networks, ovarian cancer cell line data from GSE47856 [25] and patient data from GSE15622 [26] and GSE146965 [27] were analyzed. First, cisplatin-sensitive CH1, PA1, and A2008 (GI_50_ <5 µM) cell lines were used to determine pathway enrichment based on parametric gene set enrichment analysis (PGSEA) scores [28]. Through the random forest feature selection method [29], the most relevant pathways enriched during cisplatin treatment were established. WNT/β-catenin and SHP2 signaling were identified as the top enriched pathways in all three cell lines (Fig. 1A). Although these pathways were enriched in highly sensitive ovarian cancer cell lines, their enrichment was inconsistent in relatively less sensitive ovarian cancer cell lines (Fig. 1B). Furthermore, single sample GSEA (ssGSEA) [30] comparisons for 23 cisplatin-treated (GSE146965) and six carboplatin-treated (GSE15622) ovarian cancer patients (pre- and -post-treatment) revealed significant enrichment of WNT/β-catenin and SHP2 signaling pathways (Fig. 1C). To explore whether components of WNT/β-catenin signaling are regulated during cisplatin treatment, ES2 cells were treated with cisplatin and Ser 9 phosphorylation on GSK3β was measured. As expected, Ser 9 phosphorylation on GSK3β increased at higher cisplatin concentrations (Fig. 1D). In addition, upregulation of ERK1/2 (Fig. 1D) and AKT (Fig. 1E) phosphorylation was observed at higher cisplatin concentrations.

### 2.2 Cisplatin sensitivity prediction model using TabNet

**Fig. 2:**
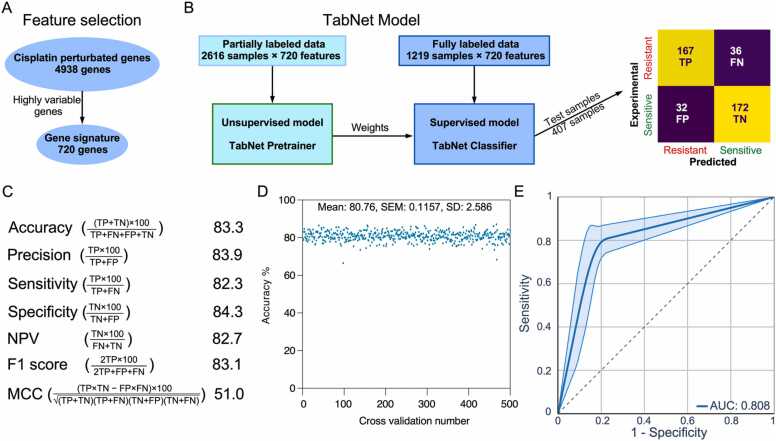
Development of the TabNet prediction model. (A) Features were selected using cisplatin-perturbation gene signatures based on the combination of cell lines and ovarian cancer patients’ data. (B) A TabNet binary classification was created by incorporating unlabeled data with the labeled data. First, an unsupervised model was built; then, its weights were added to construct the supervised binary classification model. The model was tested using 407 samples. (C) Scores were calculated from the confusion matrix using corresponding formulas. (D) Accuracy values from 500 different TabNet models. (E) The receiver operating characteristic (ROC) curves were generated using data from 500 different TabNet models.

Cisplatin-induced changes in gene expression were subsequently determined to develop cisplatin sensitivity prediction models. Gene expression changes upon cisplatin treatment are highly heterogeneous within cell lines and in ovarian cancer patients. Thus, gene expression changes upon cisplatin treatment in highly sensitive cell lines (CH1, PA1, and A2008) were combined with gene expression changes in patients displaying upregulation/downregulation of WNT/β-catenin and SHP2-signaling. The merging of both sets of gene expression change data collected highly variable genes, which resulted in the identification of a 720-gene signature (Fig. 2A). With this gene signature, machine learning models for predicting cisplatin sensitivity were developed and tested. TabNet, a recently developed interpretable canonical deep tabular data learning algorithm, was utilized [31]. TabNet incorporates unlabeled data into the supervised model, which improves prediction performance. TabNet pretrainer was fed with 2616 samples that contained both labeled and unlabeled gene expression data to develop an unsupervised model. Weights from the unsupervised model along with labeled data were then fed to create the prediction model (Fig. 2B). The model was tested using 407 samples, of which 339 (83.3 %) were precisely predicted (Fig. 2B). As shown in Fig. 2C, the model’s accuracy, precision, sensitivity, specificity, negative predicted value (NPV) and F1 score exceeded 80 %. Furthermore, the Matthew's correlation coefficient (MCC) value was high. The algorithm was then evaluated by drawing a 20 % train sample from the entire dataset 500 times (500 different samples), with the corresponding 80 % of samples used to develop the model (500 models). Consistent predictive performance was observed (Fig. 2D), with an average AUC of 0.808 (Fig. 2E).

### 2.3 The TabNet model’s predictive performance was considerably superior to that of other machine learning algorithms

**Fig. 3:**
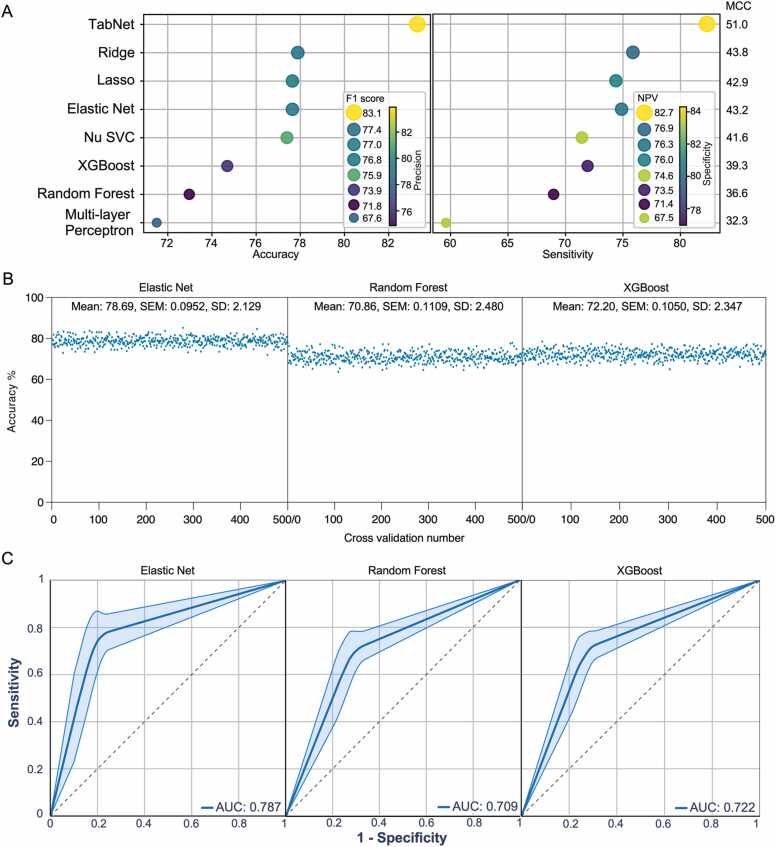
TabNet outperforms other machine learning algorithms. (A) Accuracy (left, x-axis), precision (left, color), F1 score (left, size), sensitivity (right, x-axis), specificity (right, color), NPV (right, size), and MCC (right side) scores were calculated using respective confusion matrices. (B) Accuracy values from 500 different models. (E) The ROC curves were generated using data from 500 different models.

The TabNet model was next compared to a panel of machine learning algorithms. As shown in Supplementary table 1, with some exceptions, the majority of algorithms displayed more than 70 % accuracy, denoting that the identified gene signature may efficiently explain cisplatin sensitivity. While TabNet produced the top-performing model, other algorithms, such as ridge, lasso, elastic net, Nu SVC, XGBoost, and random forest, also displayed a comparable predictive performance (Fig. 3A). Elastic net, XGBoost, and random forest algorithms were also tested using 500 different samplings, in a similar method as mentioned above for Fig. 2D. Although all three algorithms exhibited considerably consistent predictive performance as expected (Fig. 3B), none outperformed TabNet (Fig. 3C).

### 2.4 Feature importance includes several antiapoptotic genes

**Fig. 4:**
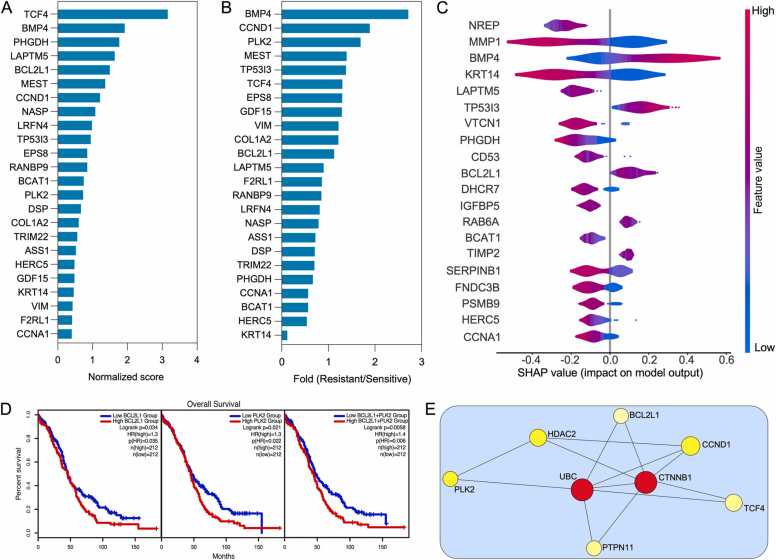
Important features explaining cisplatin resistance. (A) Important features were identified using the built-in TabNet application and SHAP (for the elastic net, random forest, and XGBoost). The figure shows normalized scores for the top features of all four models. (B) Differential expression of the top 24 features in the RNAseq data of TCGA ovarian cancer patients. Sensitivity was predicted by all four models. (C) SHAP values were calculated using the TCGA patient samples. (D) Overall survival analysis using GEPIA2. (E) Interactions between selected proteins was mapped using NetworkAnalyst 3.0.

Next, important features that contribute to the prediction of cisplatin sensitivity were discerned. The built-in feature importance function was used for the TabNet model, while SHAP values [32] were employed for the elastic net, random forest, and XGBoost models. A combined analysis of the top 100 important features identified several important genes (Fig. 4A), which are previously reported as epithelial to mesenchymal transition (EMT)-related genes (VIM, DSP, KRT14, and COL1A2), cell cycle regulators (PLK2, CCNA1, and CCND1), apoptosis regulators (BCL2L1, BMP4, and GDF15), and proteins involved in cell invasion and migration (MEST, TP53I3, F2RL1, and EPS8) [33], [34], [35], [36], [37], [38]. The generated models (TabNet, elastic net, random forest, and XGBoost models) were then used to predict cisplatin sensitivity in TCGA ovarian cancer RNAseq data. Ultimately, 92 TCGA ovarian cancer samples (46 cisplatin-sensitive and 46 cisplatin-resistant) with higher prediction probability, which were predicted by all four models, were selected. Differential expression analysis of selected features showed both upregulation and downregulation of genes in cisplatin-resistant samples (Fig. 4B). Notably, several genes involved in cell survival, such as CCND1, PLK2, TCF4, BCL2L1, etc., were found to be upregulated. SHAP values calculated within this predicted sample group also suggested the importance of a group of similar genes (Fig. 4C). In addition, differential gene expressions that were considered important features were further analyzed to discern whether they have any prognostic significance. Gene Expression Profiling Interactive Analysis (GEPIA2 [39]) was employed to perform an overall survival analysis for each gene. Only four (BCL2L1, PLK2, BCAT1, and PHGDH) out of 24 genes achieved a log-rank p-value of < 0.05 (Fig. 4D and Supplementary figure 1). Lower expression of BCAT1 and PHGDH1 (Supplementary Fig. 1B) and higher expression of BCL2L1 and PLK2 (Fig. 4D) were all associated with poor survival. Furthermore, a combined BCL2L1 and PLK2 expression (Fig. 4D) had a better hazard ratio than any other combination (Supplementary figure 1B). Additionally, we performed an interaction analysis using NetworkAnalyst 3.0 [40] showing interaction network between BCL2L1, PLK2 and β-catenin (Fig. 4E)**.**

### 2.5 Inhibitors targeting BCL2L1, PLK2, and TCF4 display synergy with cisplatin

**Fig. 5:**
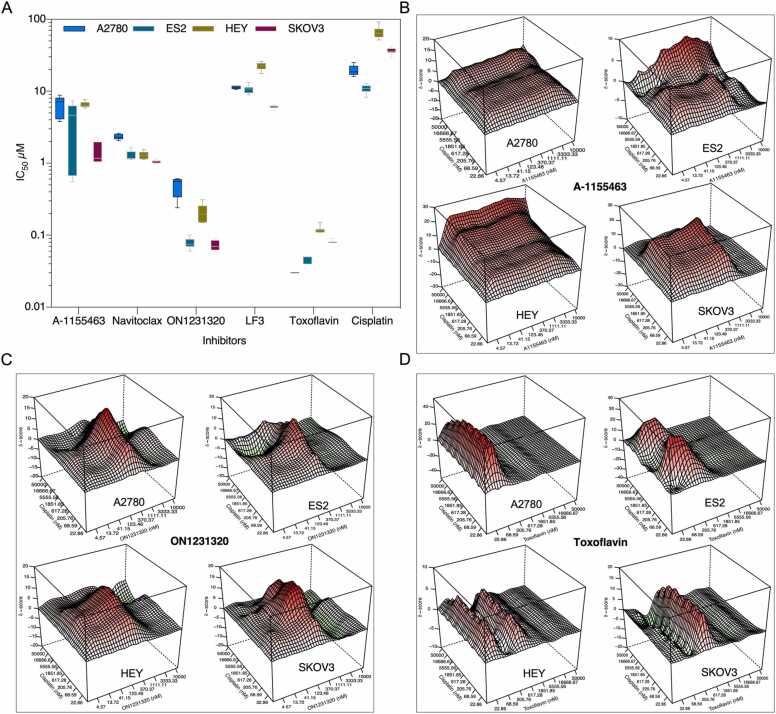
The synergy between cisplatin and BCL2L1, PLK2, and TCF4 inhibitors. (A) Cells were seeded in a 96-well plate prior to adding 8 different concentrations of each drug. After 48 h cell viability was measured using Cell Titer-Glo. IC_50_ values were calculated using GraphPad Prism. Box plot represents the IC_50_ values from 8 measurements. (B-D) Cells were treated with single drugs and 1:1 drug combination (8 concentrations). A full combination table was predicted using DECREASE [41] and ZIP synergy scores were calculated using Synergy Finder.

It was next hypothesized that inhibitors of pro-survival proteins identified by feature importance may play a role in ovarian cancer cell growth. A-1155463 (BCL2L1 specific), navitoclax (targets BCL2L1 and several other BCL2 family proteins), ON1231320 (PLK2 specific), and TCF4/β-catenin complex antagonist (LF3 and toxoflavin) were the inhibitors used. Four ovarian cancer cell lines displaying differential responses to cisplatin (ES2 >A2780 >SKOV3 >HEY) were treated with specific inhibitors for 48 h. All cell lines exhibited differential sensitivity to different inhibitors (Fig. 5A). Toxoflavin showed strong growth inhibition in all cell lines, implying that β-catenin may be involved in cisplatin resistance. Furthermore, all five drugs displayed synergy with cisplatin, with the effect being significantly greater in highly resistant cell lines (Fig. 5B, C, D, and Supplementary figures 2A and 2B).

## 3 Discussion

In this study, a panel of genes that can predict cisplatin sensitivity based on gene expression data was identified using pathway enrichment and drug-perturbed gene signatures. Initially, a wide range of machine learning algorithms was employed. While several of these, such as ridge, lasso, elastic net, random forest, and XGBoost displayed considerable predictive performance, TabNet demonstrated superior performance. Further analysis was also performed to identify important features and observed synergistic effects when cisplatin was used with BCL2L1, PLK2, and TCF4 inhibitors.

Cells treated with cisplatin displayed enrichment in the WNT/β-catenin pathway and SHP2 signaling (Fig. 1A-B), a phenotype that is maintained in cisplatin-treated ovarian cancer patients (Fig. 1C). Several previous studies have established a relationship between cisplatin and the WNT/ β-catenin pathway. To illustrate, cisplatin-resistant non-small cell lung cancer (NSCLC) cell line showed upregulation of β-catenin due to phosphorylation-dependent inhibition of GSK3β [42], and cisplatin-resistant hepatocellular carcinoma cells exhibited nuclear accumulation of GSK3β [43]. GSK3β is an essential component of the destruction complex that maintains a β-catenin cellular level by inducing phosphorylation-dependent degradation [44]. Stabilization of β-catenin, caused by phosphorylation-dependent inhibition of GSK3β, facilitates nuclear translocation and transcription of WNT response genes. Moreover, GSK3β is a substrate of several serine/threonine kinases including ERK and AKT [45], [46]. Cisplatin induces AKT phosphorylation (Fig. 1F), which was, as reported, maintained in cisplatin-resistant NSCLC cells with increased levels of β-catenin [47]. Furthermore, overexpression of β-catenin in oral squamous cell carcinoma (OSCC) induced cisplatin resistance [48], while reduction of β-catenin in cisplatin-resistant ovarian cancer cell line restored cisplatin sensitivity [49]. Additionally, enrichment of SHP2 signaling (Fig. 1A-C) can also potentiate the oncogenic activity of β-catenin [50]. SHP2 can activate both AKT and ERK [51], which in turn inactivate GSK3β and stabilize β-catenin. WNT/β-catenin plays a central role in cisplatin resistance, as indicated by this paper’s findings and supporting evidence from past studies on various types of cancer.

In previous research, only six to 15 genes were used to predict cisplatin sensitivity [21], [22], [23], [24]. However, due to the heterogeneous nature of cancer, patients with the same cancer subtype may express signature genes at varying levels. Hence, using a small number of genes to predict drug sensitivity may lower the predictive power. This was made evident when all four studies identified and employed different gene sets to predict cisplatin sensitivity. In this study, however, 720 genes selected through cisplatin-perturbation and pathway regulation (Fig. 2A) were employed to develop a model that may be less sensitive to heterogeneity than a model built with a small number of genes.

Both the quality of pharmacogenomic data and the number of samples are crucial for constructing a predictive model using machine learning algorithm [52]. Most studies used pharmacogenomic data from cell lines due to the availability of a large panel of cell line data. However, cell line data do not always represent clinical observation. Thus, this study not only used the limited number of cisplatin response data from the TCGA, but it also leveraged a large number of unlabeled patient data available in public domains. To this end, TabNet was employed, and its major strength is that it permits the use of unlabeled data, which improves predictive performance. Moreover, compared to a variety of machine learning algorithms, TabNet enabled this study to achieve the highest number of scores in terms of accuracy, precision, specificity, sensitivity, negative predictive value, F1 score, and Matthew correlation coefficient (Fig. 3A and supplementary table 1). Although models developed in this study performed well with the test data, models have not been tested with a large number of unknown samples and therefore, we cannot exclude the possibility that models may perform poorly with unknown datasets and may need hyperparameter tuning.

In line with the pathway enrichment data, using feature importance from multiple prediction models, BCL2L1 has been identified as a key gene mediating cisplatin resistance (Fig. 4A-C). Earlier studies revealed that β-catenin expression is required to maintain a higher level of BCL2L1 in mature T-cells and cisplatin-resistant lung adenocarcinoma cells [53], [54]. However, it remains to be determined how BCL2L1 expression is regulated in response to cisplatin in ovarian cancer. Furthermore, β-catenin was reported to be involved in the expression of PLK2 in mouse embryonic stem cells, and PLK2 was activated by dexamethasone-induced stabilization of β-catenin [55], [56]. Lower expression of BCL2L1 and PLK2 displayed favorable outcomes independently, and when combined (Fig. 4D) indicating a role of BCL2L1 and PLK2 in ovarian cancer. Additionally, inhibitors targeting BCL2L1 and PLK2 displayed synergy when combined with cisplatin (Fig. 5B-C), further linking BCL2L1 and PLK2 to the cisplatin response. Collectively, data presented in this study indicate that the β-catenin/BCL2L1 axis may play a vital role in cisplatin resistance.

## 4 Materials and methods

### 4.1 Cell line culture and inhibitors

The ovarian cancer cell line HEY was from Tebu-bio, while SKOV3 (SK-OV-3) and ES2 (ES-2) cell lines were from Sigma-Aldrich and ATCC, respectively. The A2780 cell line was kindly given by Dr. Chris D. Madsen (Lund University). Human ovarian adenocarcinoma cell line, A2780 was cultured in RPMI-1640 supplemented with 10 % FBS and 2 mM glutamine [57]. ES2 cell line, which was initially described as an ovarian clear cell adenocarcinoma cell line and later was suggested as ovarian serous adenocarcinoma origin [58], was cultured in McCoy’s 5a supplemented with 10 % FBS. High-grade ovarian serous adenocarcinoma cell line HEY was cultured in DMEM supplemented with 10 % FBS and 2 mM glutamate [59]. Ovarian serous cystadenocarcinoma cell line SKOV3 was cultured in McCoy’s 5a supplemented with 2 mM glutamate. Selective BCL2L1 inhibitor A-1155463 [60], potent BCL2, BCL2L1, and BCL2L2 inhibitor navitoclax [61], selective PLK2 inhibitor ON1231320 [62], and TCF4/β-catenin complex formation inhibitors (LF3 [63] and toxoflavin [64]) were purchased from MedChemExpress.

### 4.2 Western blotting

Cells were seeded in six-well plates a day before the addition of cisplatin. Cisplatin was dissolved in water and added 24 h before lysis. Cell lysates were mixed with 2X SDS sample loading buffer, and 10 μg total proteins from each lysate were separated on SDS-PAGE gels. The separated proteins were then transferred to polyvinylidene difluoride (PVDF) membranes and probed with specific antibodies. Anti-phospho-ERK1/2 and anti-phospho-AKT antibodies were from Cell Signaling Technology. The anti-GSK3β-Ser9 antibody was from Thermo Fisher Scientific, while the anti-ACTB antibody was from Santa Cruz Biotechnology.

### 4.3 Pharmacogenomic data

Gene expression data were obtained from gene expression omnibus (GEO) [65], cBioPortal [66], and Dependency Map (DepMap) portal [67]. Drug sensitivity data were collected from the Genomics of Drug Sensitivity in Cancer (GDSC) [68], the PharmacoDB [69], and the cloud-based orchestration platform for reproducing multimodal data (ORCESTRA) [70]. Cell line with GI_50_ < 5 µM or IC_50_ < 5 µM of cisplatin was considered as sensitive cell line. TCGA annotations were used for ovarian cancer patient data.

### 4.4 Machine learning models

To develop binary classification models, gene expression data (cell lines and ovarian cancer patients) from different platforms were combined. Both microarray and RNAseq data were used, and all data were log2-normalized. In all experiments, 80 % of the data were used for training (and validation, if needed), while 20 % were used for testing. Jupyter Notebook was employed to execute Python commands. For TabNet, the PyTorch framework was applied, and the model was developed on CUDA. All other machine learning programs were run on the CPU. The majority of machine learning models used are available at sklearn (scikit-learn.org). Logistic regression, passive-aggressive classifier, linear perceptron classifier and stochastic gradient descent (SGD) classifier were used from sklearn.linear_model with different regularization parameters. Bernoulli Naive Bayes, complement Naive Bayes classifier, Gaussian Naive Bayes classifier, multinomial Naive Bayes classifier were from sklearn.naive_bayes. The decision tree classifier and Extra tree classifier were from sklearn.tree. From sklearn.ensemble several tree-based models including ADA boost classifier, bagging classifier, extra trees classifier, gradient boosting classifier, histogram-based gradient boosting classifier, and random forest classifier were used. The nearest centroid classifier, k-nearest neighbors classifier, and radius neighbors classifier were from sklearn.neighbors. Support vector machine algorithms linear support vector classifier, support vector classifier, and nu-support vector classifier were from sklearn.svm. Other models include the Gaussian process classifier (sklearn.gaussian_process), multi-layer perceptron (MLP) classifier (sklearn.neural_network), calibrated classifier CV (sklearn.calibration) with Gaussian Naive Bayes (sklearn.naive_bayes) estimator, and sklearn-wrapped version of Cat boost classifier [71], Light GBM (LGBM) classifier [72] and XGboost classifier [73].

### 4.5 Drug sensitivity and drug synergy

To measure drug sensitivity and drug synergy, A2780, ES2, HEY, and SKOV3 cells were seeded in a 96-well plate. Drugs were diluted using serial dilutions with a 10 µM highest concentration for A-1155463, ON1231320, and navitoclax, and 50 µM highest concentration for cisplatin, LF3, and toxoflavin. Eight different concentrations were used to treat cells for 48 h. Cell Titer-Glo (Promega) was used to measure cell viability following drug treatment. For synergy, 1:1 drug combinations were utilized, and then full combination (8 ×8) values were predicted by DECREASE [41]. Synergy was measured by Synergy Finder [74], and ZIP synergy scores were employed to visualize synergy plots.

### 4.6 Tools and data availability

All raw data are available upon request to the corresponding author. Several tools and databases used for data collection and processing are publicly available. Random forest feature selection: sklearn.feature_selection.SelectFromModel (scikit-learn.org); ORCESTRA: www.orcestra.ca.

PGSEA: rdrr.io/bioc/PGSEA; DECREASE: decrease.fimm.fi; DepMap: www.depmap.org; Synergy Finder: synergyfinder.fimm.fi; cBioPortal: www.cbioportal.org; PharmacoDB: www.pharmacodb.ca; GEO: www.ncbi.nlm.nih.gov/geo; GDSC: www.cancerrxgene.org; Network Analyst 3.0: www.networkanalyst.ca.

## CRediT authorship contribution statement

Ahmad Nasimian: Performed experiments, analyzed data, and wrote the manuscript, Mehreen Ahmed: Performed experiments, analyzed data, and wrote the manuscript, Ingrid Hedenfalk: Analyzed data and wrote the manuscript, Julhash U. Kazi: Performed experiments, analyzed data, supervised the research, designed the study, and wrote the manuscript.

## Conflicts of interest

The authors declare no conflicts of interest.
