# Supplementary material for: A deep tabular data learning model predicting cisplatin sensitivity identifies BCL2L1 dependency in cancer
Source: Comput Struct Biotechnol J. 2023 Jan 16;21:956–64. doi: 10.1016/j.csbj.2023.01.020 (PMC9876747; doi:10.1016/j.csbj.2023.01.020)

Supplementary figure 1. Overall survival. Overall survival was analyzed using GEPIA2.

A

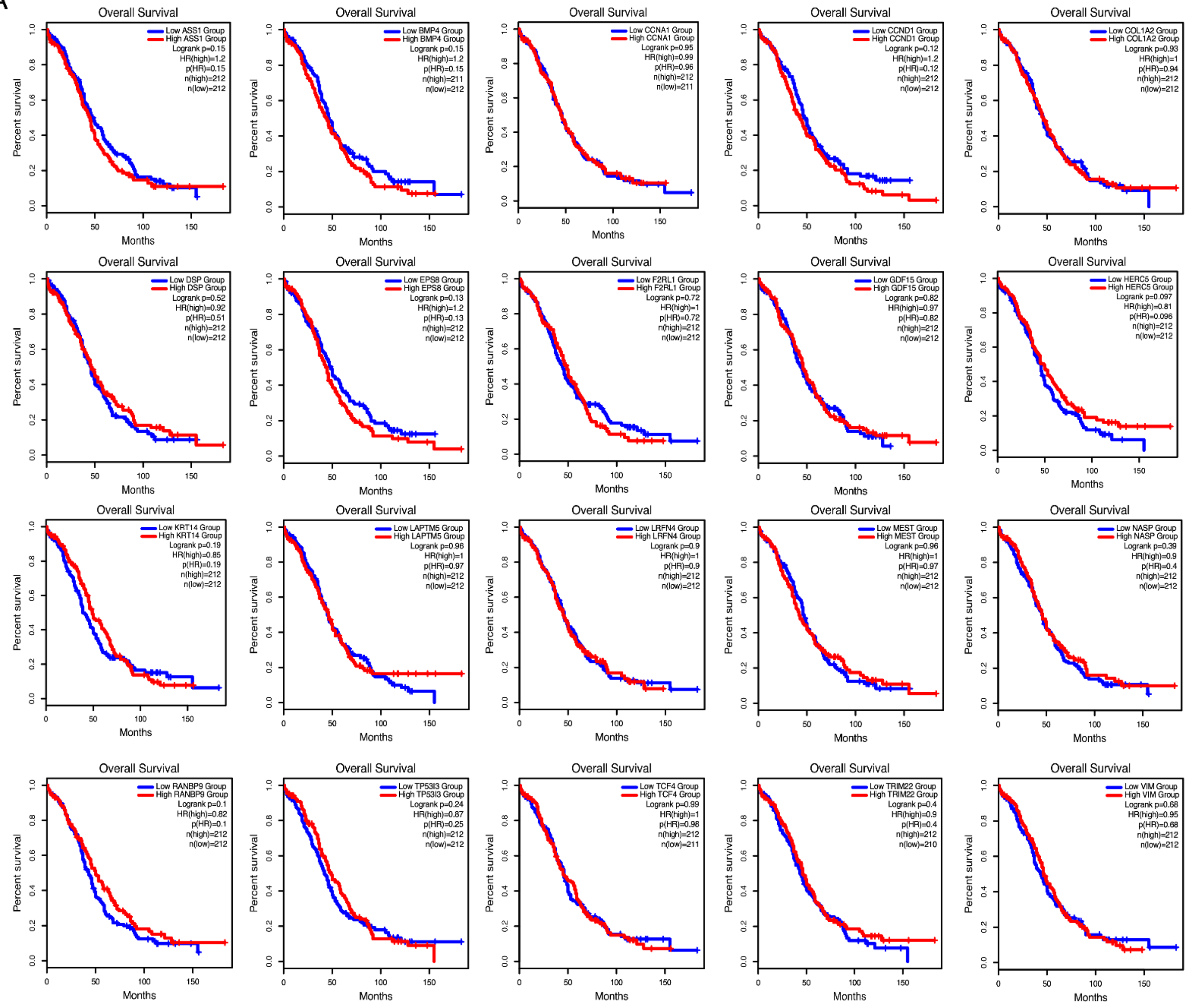

B

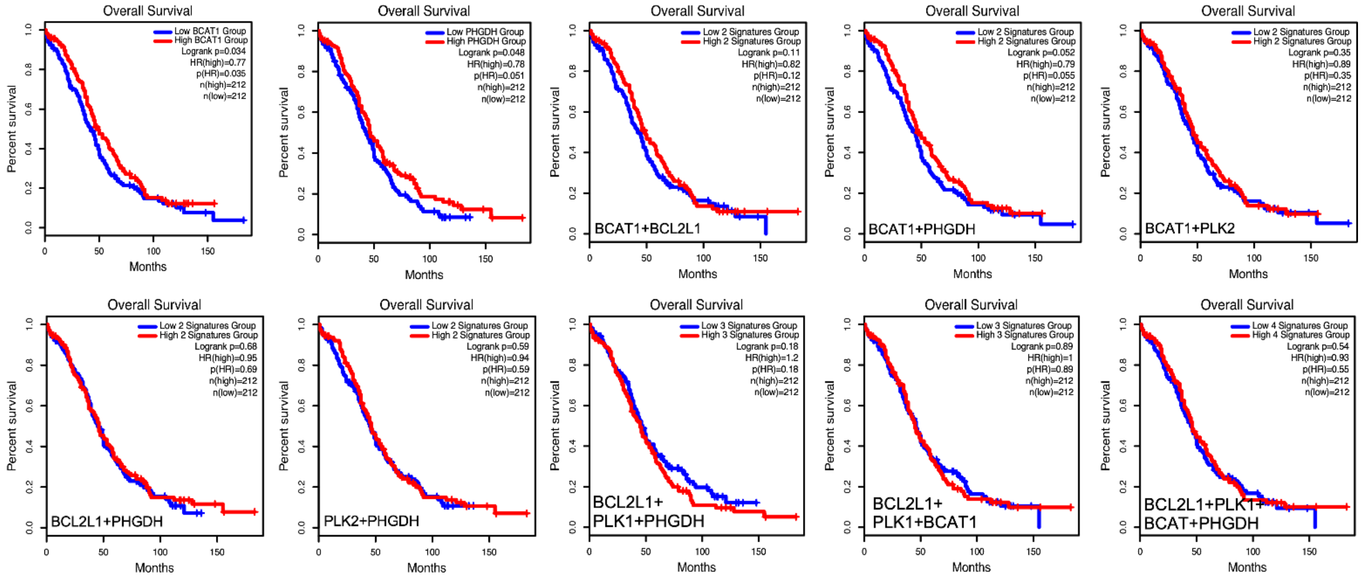

Supplementary figure 2. Drug synergy. Cells were treated with single drugs and 1:1 drug combination (8 concentrations). A full combination table was predicted using DECREASE and synergy was calculated using Synergy Finder.

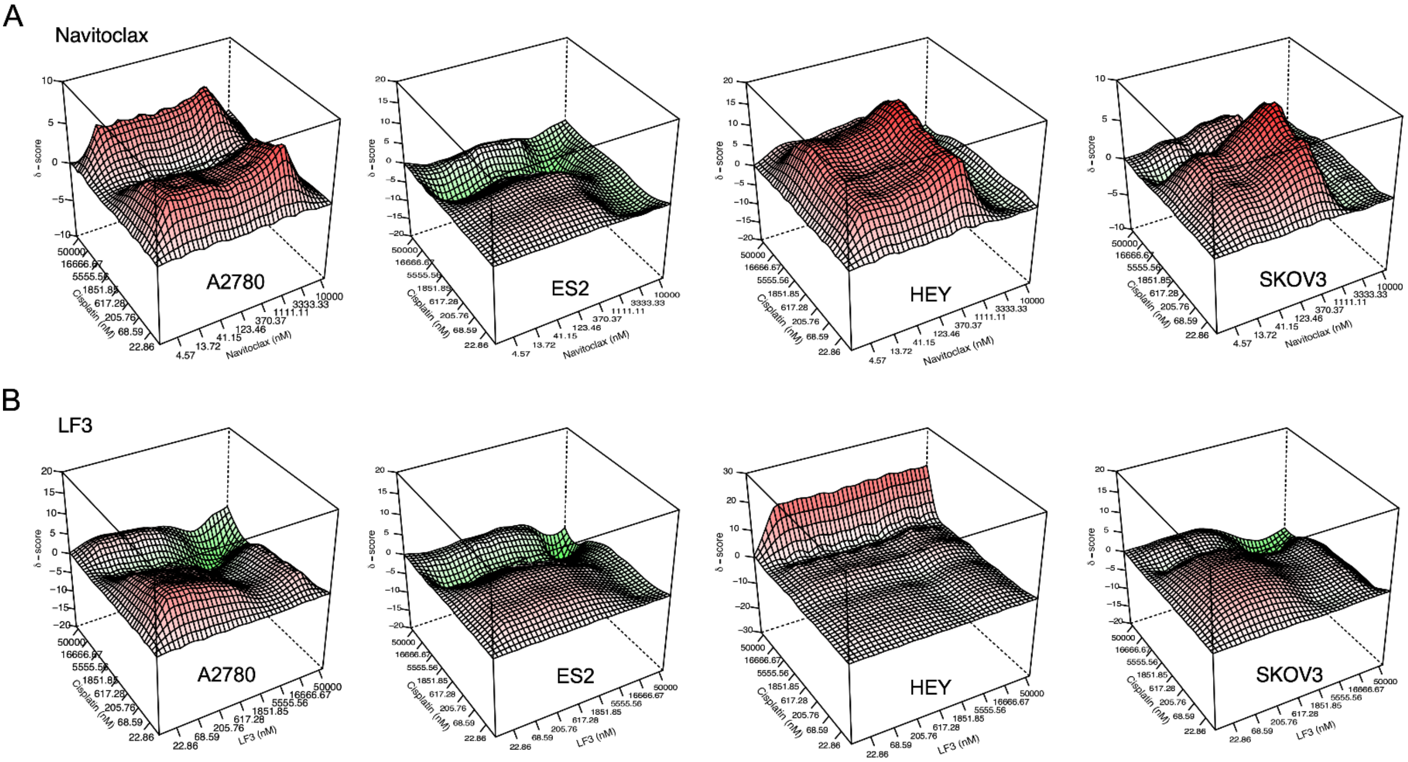

Supplement: Supplementary file 1 — Supplementary material. [file mmc1.pdf]
