# Supplementary material for: A deep tabular data learning model predicting cisplatin sensitivity identifies BCL2L1 dependency in cancer
Source: Comput Struct Biotechnol J. 2023 Jan 16;21:956–64. doi: 10.1016/j.csbj.2023.01.020 (PMC9876747; doi:10.1016/j.csbj.2023.01.020)
Supplement: Supplementary file 2 — Supplementary material. [file mmc2.pdf]

Supplementary Table 1. Predictive performance for different machine learning algorithms

Classifier	Parameters	Accuracy	Precision	Sensitivity	Specificity	NPV	F1 score	MCC
LogisticRegression	max_iter=1000, solver="saga", random_state=18	77.9	79.0	75.9	79.9	76.9	77.4	43.8
	penalty='l1', max_iter=1000, solver="saga", random_state=18	77.6	79.5	74.4	80.9	76.0	76.8	42.9
	penalty='elasticnet', max_iter=1000, solver="saga", l1_ratio=0.5, random_state=18	77.6	79.2	74.9	80.4	76.3	77.0	43.2
PassiveAggressiveClassifier	random_state=18	77.6	80.8	72.4	82.8	75.1	76.4	42.2
Perceptron	random_state=18	74.9	83.9	61.6	88.2	69.8	71.0	36.1
	penalty='l2', random_state=18	63.6	89.9	30.5	96.6	58.3	45.6	18.6
	penalty='l1', random_state=18	71.5	66.4	86.7	56.4	81.0	75.2	43.1
	penalty='elasticnet', random_state=18	75.4	72.3	82.3	68.6	79.5	77.0	44.6
SGDClassifier	random_state=18	74.0	88.2	55.2	92.6	67.5	67.9	33.5
	penalty='l1', random_state=18	75.9	79.7	69.5	82.4	73.0	74.2	39.5
	penalty='elasticnet', random_state=18	75.2	85.4	60.6	89.7	69.6	70.9	36.1
	loss='log_loss', random_state=18	74.9	82.6	63.1	86.8	70.2	71.5	36.6
	loss='log_loss', penalty='l1', random_state=18	77.4	81.4	70.9	83.8	74.3	75.8	41.4
	loss='log_loss', penalty='elasticnet', random_state=18	70.0	87.2	46.8	93.1	63.8	60.9	27.9
	loss='modified_huber', random_state=18	72.0	86.8	51.7	92.2	65.7	64.8	30.8
	loss='modified_huber', penalty='l1', random_state=18	78.4	78.0	78.8	77.9	78.7	78.4	45.5
	loss='modified_huber', penalty='elasticnet', random_state=18	74.0	70.5	82.3	65.7	78.8	75.9	43.1
GaussianProcessClassifier		67.8	65.3	75.9	59.8	71.3	70.2	33.4
BernoulliNB		50.4	50.1	88.2	12.7	52.0	63.9	1.9
ComplementNB		66.3	64.9	70.9	61.8	68.1	67.8	29.8
GaussianNB		63.4	60.1	79.3	47.5	69.8	68.4	28.9
CalibratedClassifierCV	GaussianNB()	43.0	43.7	49.3	36.8	42.1	46.3	-16.1
	GaussianNB(), method="isotonic"	59.2	56.9	74.9	43.6	63.6	64.7	20.5
MultinomialNB		66.3	64.9	70.9	61.8	68.1	67.8	29.8
DecisionTreeClassifier	random_state=18	61.9	61.4	63.5	60.3	62.4	62.5	21.8
ExtraTreeClassifier	random_state=18	59.2	59.1	59.1	59.3	59.3	59.1	16.9
AdaBoostClassifier	n_estimators=100, random_state=18	63.6	64.0	62.1	65.2	63.3	63.0	23.8
BaggingClassifier	n_estimators=100, random_state=18	75.4	77.2	71.9	78.9	73.9	74.5	40.0
ExtraTreesClassifier	random_state=18	71.7	72.9	69.0	74.5	70.7	70.9	35.3
GradientBoostingClassifier	random_state=18	70.0	72.1	65.0	75.0	68.3	68.4	32.2
HistGradientBoostingClassifier	random_state=18	72.2	73.4	69.5	75.0	71.2	71.4	36.0
RandomForestClassifier	n_estimators=150, max_depth=16, random_state=18	73.0	74.9	69.0	77.0	71.4	71.8	36.6
CatBoostClassifier	iterations=100, learning_rate=0.1, random_state=18	71.5	73.8	66.5	76.5	69.6	69.9	34.3
LGBMClassifier	random_state=18	72.2	74.2	68.0	76.5	70.6	71.0	35.5
XGBClassifier	n_estimators=150, learning_rate=0.1, random_state=18	74.7	76.0	71.9	77.5	73.5	73.9	39.3
KNeighborsClassifier	n_neighbors=250	63.6	60.4	78.8	48.5	69.7	68.4	29.0
NearestCentroid		62.2	60.2	71.4	52.9	65.1	65.3	24.2
RadiusNeighborsClassifier	radius=100	52.3	51.4	79.3	25.5	55.3	62.4	6.9
MLPClassifier	hidden_layer_sizes=(720,512,256), max_iter=1000, random_state=18	71.5	78.1	59.6	83.3	67.5	67.6	32.3
LinearSVC	random_state=18, dual=False	73.5	75.1	70.0	77.0	72.0	72.4	37.4
	penalty='l1', random_state=18, dual=False	75.7	77.7	71.9	79.4	74.0	74.7	40.2
NuSVC	random_state=18	77.4	81.0	71.4	83.3	74.6	75.9	41.6
SVC	random_state=18	73.5	76.0	68.5	78.4	71.4	72.0	36.9
	kernel="linear", random_state=18	73.7	75.0	70.9	76.5	72.6	72.9	38.0
	kernel="poly", degree=15, random_state=18	75.7	77.4	72.4	78.9	74.2	74.8	40.4
	kernel="sigmoid", random_state=18	50.9	50.6	58.1	43.6	51.1	54.1	1.9
